# MicroRNA-302a is involved in folate deficiency-induced apoptosis through the AKT-FOXO1-BIM pathway in mouse embryonic stem cells

**DOI:** 10.1186/s12986-020-00530-3

**Published:** 2020-12-07

**Authors:** Yan Liang, Dingding Cao, Yuanyuan Li, Zhuo Liu, Jianxin Wu

**Affiliations:** 1 Department of Pediatric Respiratory, Shandong Provincial Hospital Affiliated to Shandong First Medical University, Jinan, Shandong, 250021 China; 2 Department of Biochemistry and Immunology, Capital Institute of Pediatrics, Beijing, 100020 China

**Keywords:** miR-302a, Akt/FOXO1/Bim pathway, Folate deficiency, Cell apoptosis

## Abstract

**Background:**

Our previous study had shown that microRNA (miR)-302a played a key role in folate deficiency-induced apoptosis in mouse embryonic stem cells. However, details regarding the mechanism remain unclear. Transcription factors (TFs) and miRNAs are two key elements in gene regulation. The aim of this study is to construct the TF-miRNA gene regulation network and demonstrate its possible mechanism.

**Methods:**

The TF-miRNA gene regulation network was constructed via bioinformatics methods. Chromatin immuno-coprecipitation PCR was selected to confirm the binding between miR-302a and TF. mRNA and protein levels were detected by Real-time quantitative PCR and western blotting. TargetScan prediction and Dual-Luciferase Reporter Assay system were used to confirm whether the miRNA binded directly to the predicted target gene.

**Results:**

FOXO1 and miR-302a were selected as the key TF and miRNA, respectively. FOXO1 was confirmed to bind directly to the upstream promoter region of miR-302a. Real-time quantitative PCR and immunoblotting showed that in folate-free conditions, miR-302a and AKT were down regulated, while FOXO1 and Bim were up-regulated significantly. Additionally, treatment with LY294002 inhibitor revealed the involvement of the Akt/FOXO1/Bim signaling pathway in folate deficiency-induced apoptosis, rather than the ERK pathway. Finally, TargetScan prediction and double luciferase reporting experiments illustrated the ability of miR-302a to target the Bim 3′UTR region.

**Conclusion:**

The involvement of miR-302a in folate deficiency-induced apoptosis through the AKT-FOXO1-BIM pathway in mESCs is a unique demonstration of the regulation mechanism of nutrient expression in embryonic development.

## Introduction

Folate is a water-soluble vitamin and a cofactor for the transportation of one-carbon units in DNA biosynthesis, as well as in hundreds of methylation reactions [1, 2]. Few studies investigated whether and how folate deficiency affects the normal development of a growing embryo [3]. The expression of micro RNA (miRNA) can be regulated by epigenetic mechanisms, such as DNA methylation or histone modifications [4]. Regulatory transcription factors (TFs) control miRNA expression, working at the level of transcription initiation by directly binding to genomic DNA targets, resulting in miRNA activation or repression. TFs and miRNAs are two key elements in gene regulation. The relationships between TFs, miRNAs, and target genes are extremely complex and play an important role in the pathogenesis of diseases [5]. Folate status can influence miRNA profiles via alteration of DNA methylation of genes coding for miRNA, or upstream genes [2, 6], however, the mechanism involved remains unclear.

Our preliminary research revealed the role of miR-302a in the regulation of mESC proliferation, apoptosis and cell cycle phase [7]. In the present study, we aimed to characterize the mechanism of folate deficiency-induced cell apoptosis in mouse embryonic stem cell (mESCs). Therefore, bioinformatics analyses were used to construct a TF-miRNAs-gene network associated with folate deficiency. FOXO1 was the key TF regulating miR-302a expression, while miR-302a was the core miRNA in the network. Chromatin immunoprecipitation (ChIP)-PCR confirmed that FOXO1 directly binds to and negatively regulates the expression of miR-302a. We sought to determine the involvement of the Akt/FOXO1/Bim signaling pathway in folate deficiency-induced apoptosis. Subsequently, we discovered that miR-302a could bind directly to Bim 3′-UTR, negatively regulating Bim expression. These observations suggested that both FOXO1 and miR-302a regulate the same target gene to form a TF-miRNA-gene loop. This report will provide the scientific basis for the future research on nutrients involved in early embryonic development. These findings are reasonable and contribute to understand why and how folate deficiency induces apoptosis.

## Materials and methods

### TF-miRNA-gene network

In our preliminary research, qRT-PCR confirmed that let-7a and miRNAs 15a, 15b, 16, 29a, 29b, 34a, 130b, 125a-5p, 124, 290, and 302a were differentially expressed in folate-deficient mESCs [7]. Furthermore, TF analysis (Tfscan) illuminated the role of transcription factors in regulating these 12 miRNAs. First, the sequences of miRNA precursor genes were searched. Jemboss software was then used to discover gene-TF relationships by determining the correlations between gene and TF sequences. The property relationship between a TF and a microRNA can be used to construct the adjacency matrix A = [$a_{ij}$], which represents the relationship weight between miRNA *i* and transcription factor *j*. Next, the TF regulation network (TF-miRNA-gene network) was built using the interactions between genes and TFs. The network’s core TF was the most important central factor and had the biggest degree of distribution [8, 9]. Pearson correlation analysis was used to measure TF regulatory ability by calculating the correlation between TFs and the genes they regulate, as well as correlations with other genes regulated by the same factors [8]. The core TF was the one with the highest degree of correlation in the network.

### mESC culture and treatment

mESCs and mitomycin C-treated primary mouse embryonic fibroblast (MEF) feeder cells were obtained from Beijing Stem Cell Bank (Beijing, China) and cultured in our laboratory as previously described [7]. Folate deficient mESCs were collected at predetermined time points.

### Chromatin immunoprecipitation-quantitative polymerase chain reaction (ChIP-qPCR)

ChIP was performed on folate-deficient mESCs at 0, 24, 48, and 72 h, as previously described [10, 11] using the EZ-ChIP™ Kit (Millipore). Briefly, samples were cross-linked and treated with formaldehyde. Chromatin was sheared into 400–500 bp fragments and lysed by ultrasound. The obtained fragments were divided into two aliquots, one for ChIP using a FoxO1-specific antibody (Abcam) and the other for total input sample analysis. Normal mouse IgG was used as a negative control. The target protein and its binding DNA fragment were immunoprecipitated using BiomagTM magnetic beads (Bangs Laboratories, Inc). Finally, DNA purification, protein-DNA complex decrosslinking, and separation of DNA fragments were performed. The immunoprecipitated DNA was quantified using real-time PCR, as previously described [12]. The PCR products were analyzed by agarose gel electrophoresis and visualized with ethidium bromide under ultraviolet light. Purified DNA was also analyzed by real-time PCR using Power SYBR® Green PCR Master Mix (ABI). The Ct values of each ChIP DNA fractions were normalized to the input DNA fraction Ct value for the same qPCR Assay (ΔCt) to account for chromatin sample preparation differences [13]. The % input for each ChIP fraction was calculated as Input% = 2(Ct Input-Ct ChIP) × Fd × 100%, where Fd is input dilution factor (1/20). The primers used for PCR are listed in Table 1.

**Table 1:** Primers used in qRT-PCR

	Primer	Product size (bp)
miR-302a	RT primer: 5′GTCGTATCCAGTGCGTGTCGTGGA GTCGGCAATTGCACTGGATACGACTCACCAA3′	67
	F: 5′GGGGTAAGTGCTTCCATGTT3′	
	R: 5′CAGTGCGTGTCGTGGAGT3′	
U6	RT primer: 5′CGCTTCACGAATTTGCGTGTCAT3′	89
	F: 5′GCTTCGGCAGCACATATACTAAAAT3′	
	R: 5′CGCTTCACGAATTTGCGTGTCAT3′	
AKT	F: 5′GGCTGCTCAAGAAGGACCCTAC3′	72
	R: 5′GGTGCTGCATGATCTCCTTGG3′	
FOXO1	F: 5′GTACAGCGCATAGCACCA3′	121
	R: 5′GCGACAGACAGAGTTCCC3′	
Bim	F: 5′GTTCTGCCGCCTTTCTG 3′	189
	R: 5′TTCCCCATCTGCTGCTAA 3′	
miR-302a Promoter	F: 5′GTTACCCTAATCTGTGCCATCAA3′	284
	R: 5′AAGACGCCGACTTTAGACCAC3′	
β-actin	F: 5′TTCCAGCCTTCCTTCTTG3′	182
	R: 5′GGAGCCAGAGCAGTAATC3′	

### Quantitative real-time PCR

Stem-loop reverse transcriptase (RT)-PCR was used to detect miRNA and SYBR Green real-time RT-PCR was used to detect mRNA, using the Reverse Transcription Kit (Promega) and Power SYBR® Green PCR Master Mix (ABI), respectively. Mouse U6 and β-actin served as internal controls. The fold-change for each miRNA and mRNA was calculated using the 2^−∆∆Ct^ method [14]. The primers used for RT-PCR are shown in Table 1.

### Western blot

Total protein was extracted and quantified using the BCA method. About 40 μg of protein was loaded and separated with SDS-PAGE. PVDF membranes (Milipore) were blocked and incubated with primary antibodies against AKT, p-AKT (Phospho-Akt-Ser473), FoxO1, p-FoxO1 (Phospho-FOXO1-Ser256), Bim, and β-actin, all at a dilution of 1:1000. Finally, membranes were immunoblotted with goat anti-rabbit secondary antibodies conjugated to horseradish peroxidase (1:5000). All antibody reagents were purchased from Cell Signal Technology. Bands were detected with eECL Western Blot Kit (Cwbio, Beijing, China) and quantified using Image J software (Bio-Rad).

### Cell proliferation analysis

Folate-deficient mESCs were incubated with DMSO (control) or increasing doses of LY294002 or PD98059 (10, 20, 30, 40, 50 μM) for 12, 24, 36, or 48 h. Then, cell viability was detected by CCK-8 assay (Cell Counting Kit-8, Dojindo Laboratories, Tokyo, Japan), according to the manufacturer's instructions. The details were described in our previous study [7].

### Luciferase activity assay

The 2233 bp Bim 3′UTR fragment, which contains one well conserved and two poorly conserved miR-302a binding sites, were amplified by PCR from mouse 3T3 genomic DNA. The amplified fragments were inserted into the pmiR-RB-REPORT™ vector (Ribobio, China) using the XhoI and NotI sites. Mutant 3′UTR fragments, designed to mutate AGCACTT into ACGAGTT were generated using mutation primers. Similarly, the Bim 3′UTR mutant fragment was inserted into the pmiR-RB-REPORT™ control vector at the same sites. For reporter assays, The details were previously described in our previous study [7]. All primers are listed in Table 2

**Table 2:** Primers used in Luciferase activity assay

Sequence position	Primer
M-Bim-3′UTR -F	5′CCGCTCGAGTCAGCCCAGGGTATCTTCAAGTG3'
M-Bim-3′UTR -R	5′GAATGCGGCCGCTACAGCATTATGAAACAGTAGG3'
M-Bim-mut1-F	5′ GAGTAAACGAGTTGTCTTCCACAAGATGTCTG 3'
M-Bim-mut1-R	5′ GAAGACAACTCGTTTACTCGGCATAATGGAAG 3'
M-Bim-mut2-F	5′ TGGAGGACGAGTTTCTAACCTGTGGAGAGCTG 3'
M-Bim-mut2-R	5′ AGGTTAGAAACTCGTCCTCCACCAGGCTGTGC 3'
M-Bim-mut3-F	5′ GACTGGACGAGTTTACTGTCTCAGCCCCATGG 3'
M-Bim-mut3-R	5′ GACAGTAAACTCGTCCAGTCGGGGAGACTGGC 3'

### Statistical analysis

Data are representative of three independent experiments performed in triplicate. Statistical analyses were performed using Microsoft Excel to calculate the mean and standard deviation (SD). Statistical significance was evaluated by Student's t-test, and values with a *p* < 0.05 (two-tailed analysis) were considered statistically significant.

## Results

### Mapping a distinct TF-miRNA-gene network

**Fig. 1:**
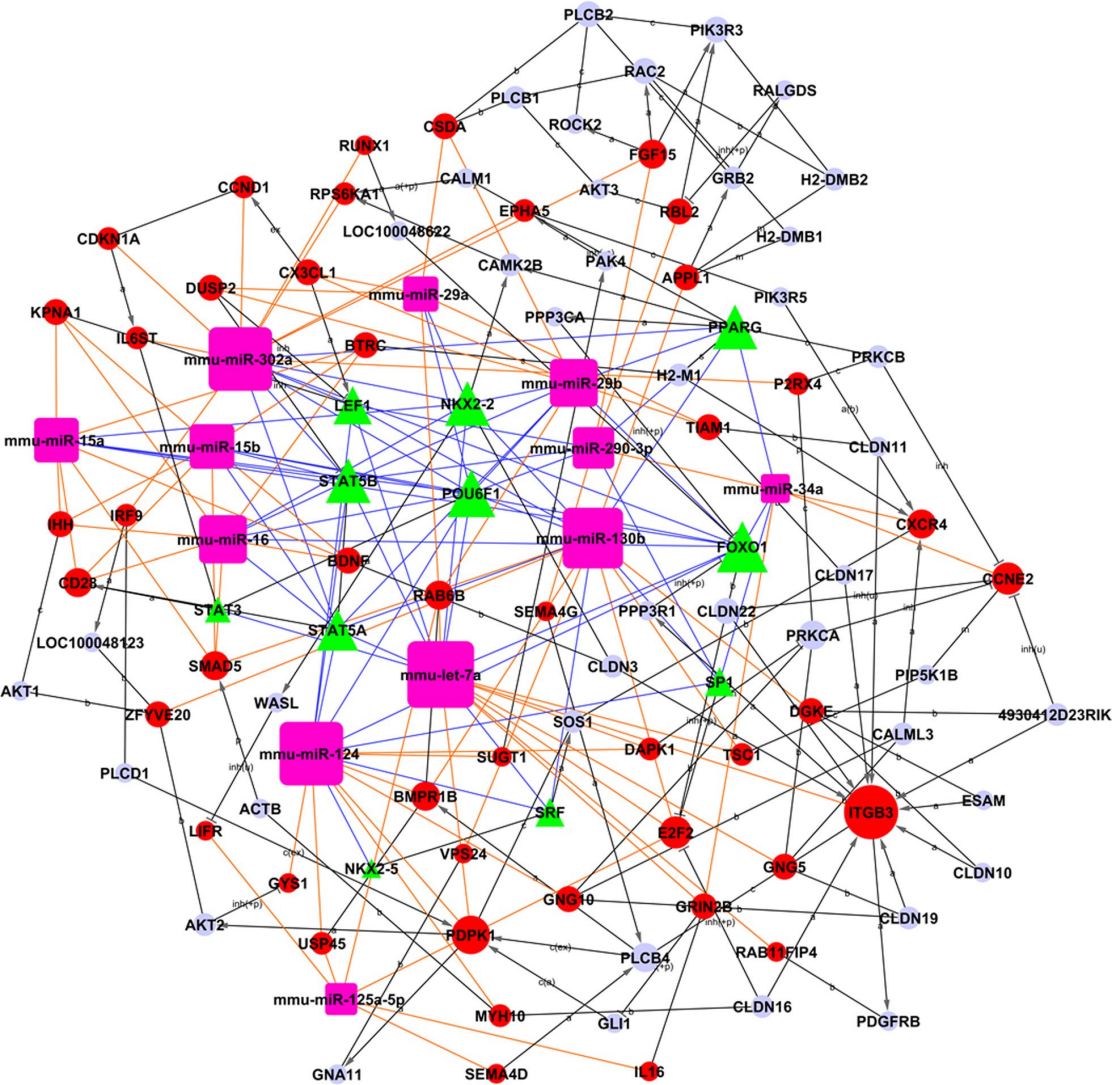
TF-miRNA-gene network. In the regulatory network, miRNAs are represented by squares, transcription factors by triangles, and interactions by one arrow shape

Network analysis showed that FOXO1 had the highest degree of correlation and regulated the largest number of genes (12), including 8 miRNAs: let-7a, miR-124a, miR-15a, miR-15b, miR-16, miR-29b, miR-302A and miR-34a. Meanwhile, let-7a had the largest number of genes involved in the regulation network (17), followed by miR-302a and miR-124a (Fig. 1). The results of a previous miRNA-GO analysis showed that miR-302a played an important role in folate deficiency-specific apoptosis. Above all, FOXO1 was determined to be the key TF in the network, and miR-302a was the core miRNA. Therefore, additional experiments focused on the possible roles of miR-302a and FOXO1 in the regulation of cell apoptosis.

### Folate deficiency alters the binding of the FOXO1 and miR-302a promoter region

**Fig. 2:**
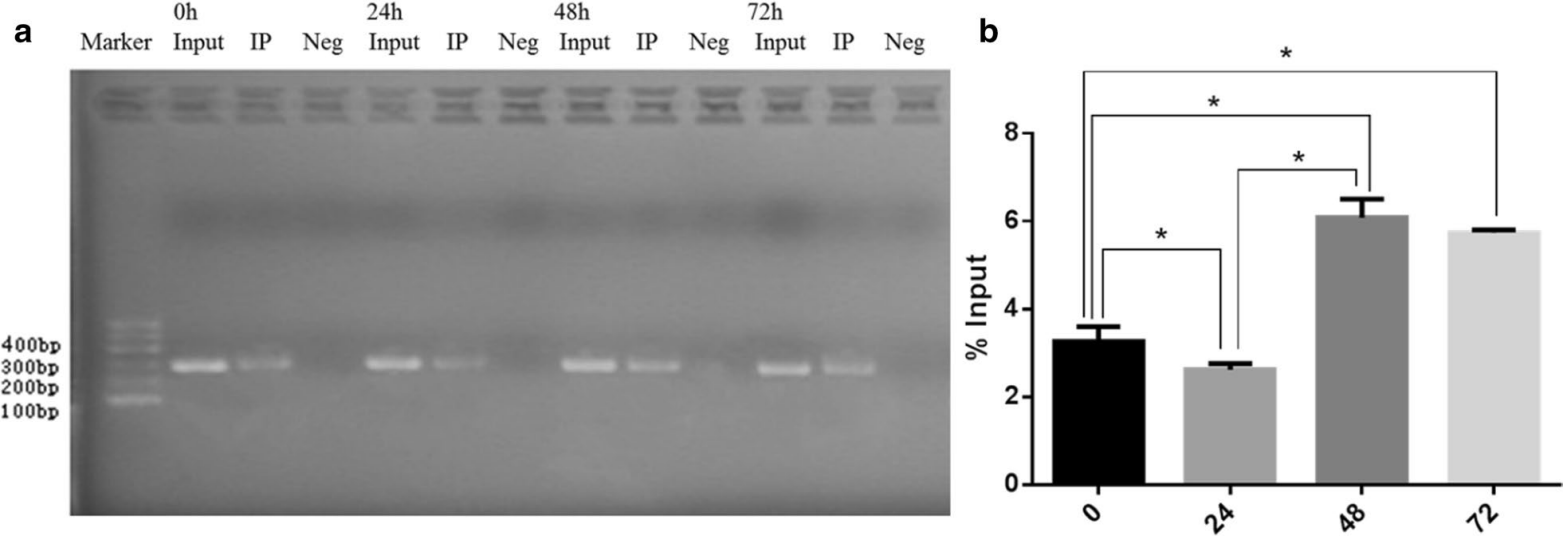
Altered bindings of the FOXO1 and miR-302a promoter region in folate deficient mESCs. **a** Bands intensity of PCR products on agarose gels. **b** ChIP enrichment were calculated among four groups: 0, 24, 48, 72 h. Values are the Means ± SD. *P < 0.05, **P < 0.01

**Fig. 3:**
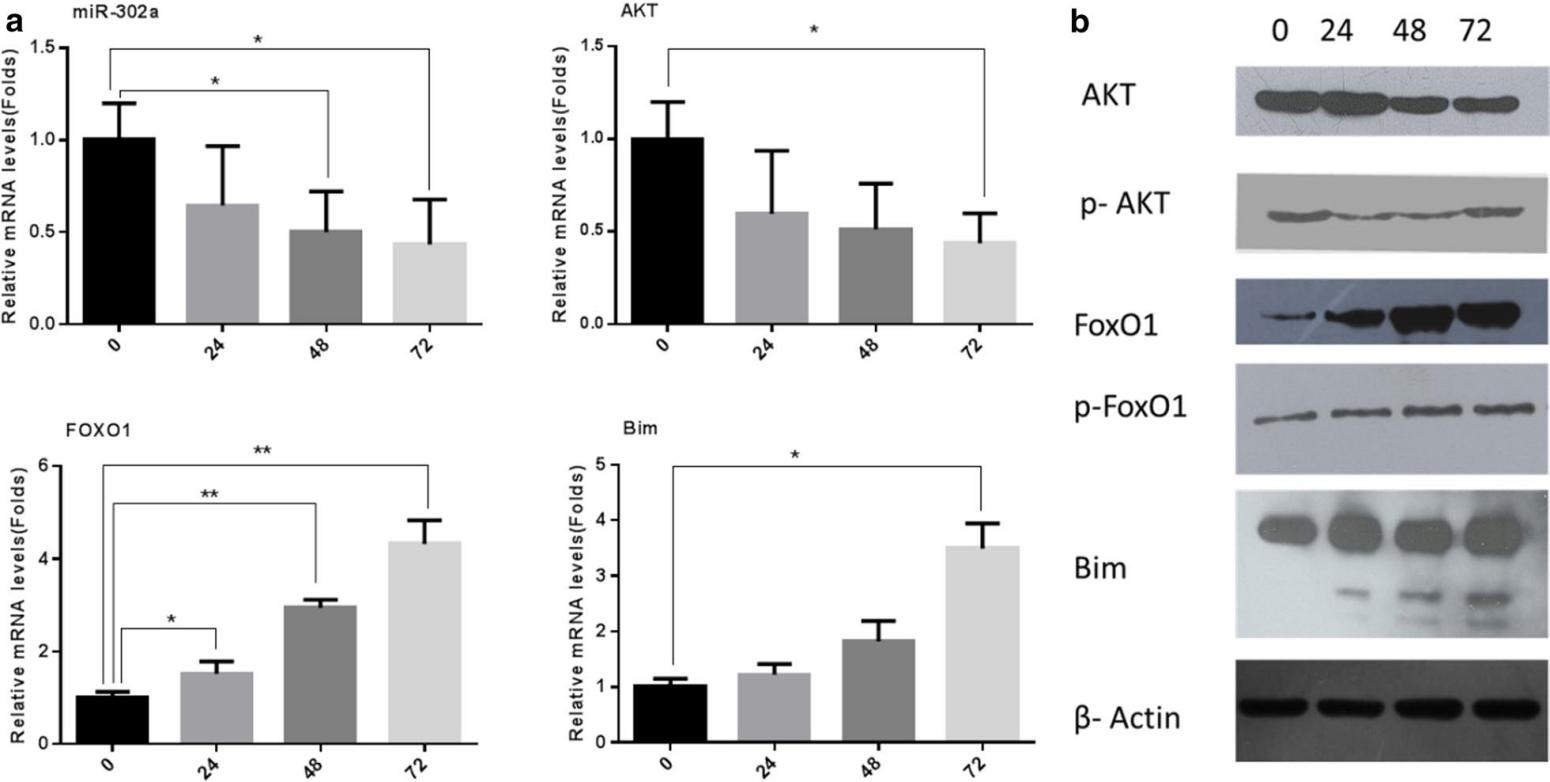
RT-PCR and Western blot quantifying miR-302a, AKT, FOXO1, Bim mRNA and protein, respectively, at different time point: 0 h, 24 h, 36 h, 48 h. Relative mRNA expression is shown as Means ± SD. β-actin and U6 are internal controls. *P < 0.05, **P < 0.01

Approximately 28 FOXO1 binding sites are found in the miR-302a promoter region. Alignment algorithms determined the best protein-DNA binding site on the miR-302a promoter region was at 904–916 bp. Bands intensity of PCR products were analyzed on agarose gels. The results confirmed the capability of FOXO1 in binding to the mouse miR-302a promoter region (Fig. 2a). The %input had the tendency to rise as folate deficiency continued. The results showed that the hybridization intensity was down-regulated at 24 h (*p* < 0.05), but up-regulated at 48 and 72 h (*p* < 0.001), which were 2.62 ± 0.14%, 6.08 ± 0.42% and 5.74 ± 0.07%, respectively. The effects of the input DNA on hybridization intensity were evident after approximately 48 h in folate-deficient culture; however, no differences were observed between samples collected at 48 and 72 h (Fig. 2b, p > 0.05). Additionally, an approximately twofold decrease of miR-302a was observed by qRT-PCR after 48 h in folate-free culture (Fig. 2a, *p* < 0.05), followed by a gradual decrease every 24 h. Conversely, FOXO1 mRNA increased fourfold at 72 h, compared with the 0 h time point (Fig. 3a, *p* < 0.001). FOXO1 protein levels were significantly up-regulated as early as 24 h in folate-free culture (Fig. 3b, 0.57 ± 0.04 vs 0.28 ± 0.03, *p* < 0.001). Thus, FOXO1 may be much more sensitive to altered folate conditions. These results indicated FOXO1 could bind with miR-302a directly and inversely correlated with miR-302a expression in folate deficient condition.

### AKT/FOXO1/Bim expressions were altered in folate deficiency mESCs

To confirm the involvement of the AKT/FOXO1/Bim signaling pathway in folate deficiency, differential expression of ATK, FOXO1, and Bim were analyzed by qRT-PCR and western blot. AKT mRNA expression levels were obviously decreased at 72 h compared to 0 h (Fig. 3a, p < 0.05), as well as AKT and p-AKT protein levels. Meanwhile, FoxO1 (1.19 ± 0.20 vs 0.28 ± 0.03, *p* < 0.001) and Bim protein levels(1.14 ± 0.06 vs 0.46 ± 0.02, *p* < 0.001) were significantly increased (Fig. 3b). These data suggest a significant inactivation of the AKT signaling pathway, while FoxO1, Bim, and Caspase-3 mRNA and protein levels were significantly increased in folate-deficient cells. Thus, folate deficiency inhibits the AKT signaling pathway, in turn activating FOXO1 and Bim expression to promote cell apoptosis.

### Folate deficiency-induced apoptosis occurs through the PI3K/Akt/FOXO1/Bim signaling pathway, not the ERK pathway

**Fig. 4:**
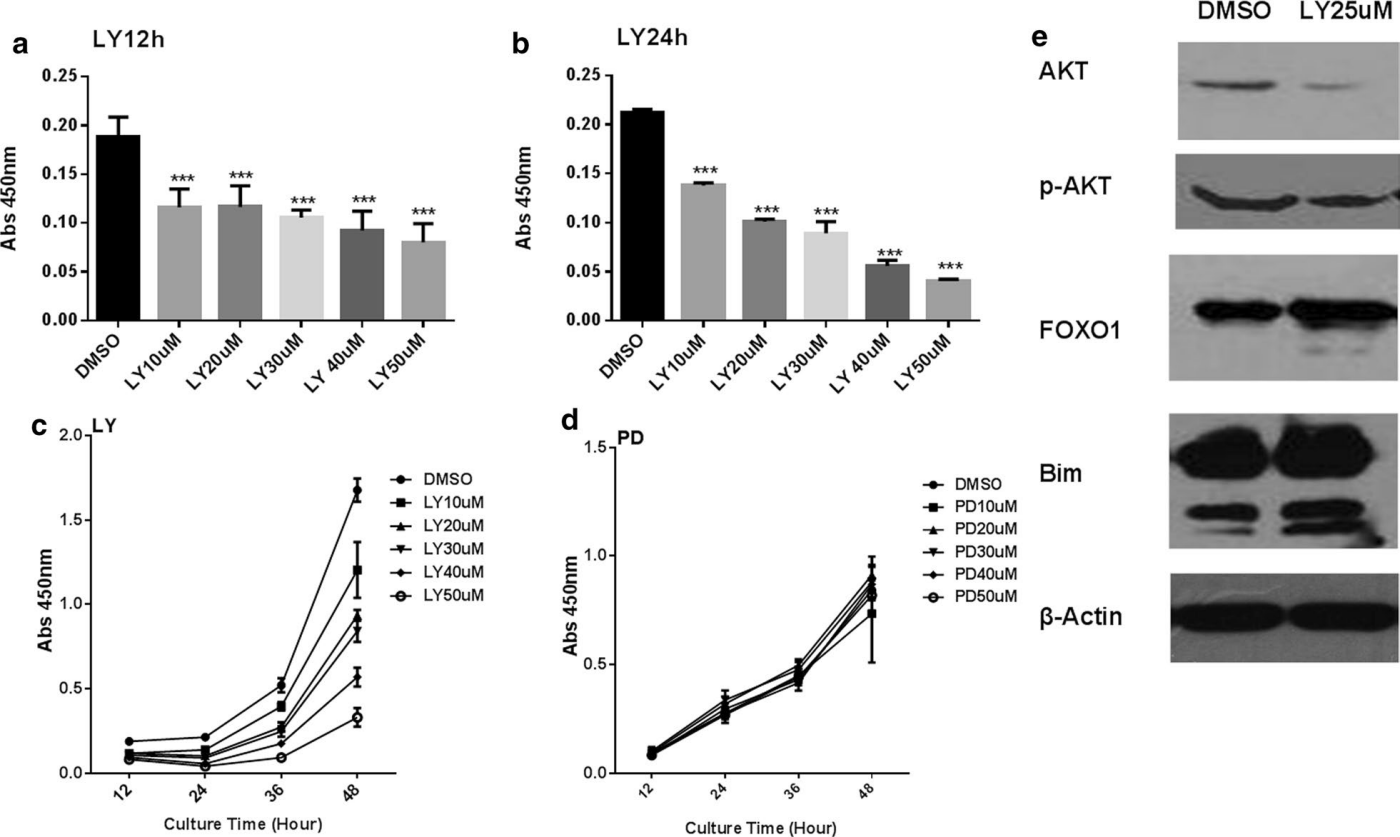
Cell viability was detected by CCK-8 assay in folate-deficient mESCs pretreated with LY294002 (LY) or PD98059 (PD). DMSO was the control. **a**–**c** Folate-deficient mESCs treated by LY294002 with different concentration shows various cell viability in dose-dependent and time-dependent manners, ***P < 0.001 versus DMSO group. **d** Cell viability of folate-deficient mESCs incubated by various concentrations of PD98059 shows no difference. **e** Proteins were analyzed in Folate-deficient mESCs treated by LY294002 25uM and DMSO control

To determine whether folate deficiency promoted cell survival through the PI3K/Akt/FOXO1 pathway, we examined the effects of the PI3K/AKT pathway inhibitor LY294002 and the MAPK-specific inhibitor PD98059. After 12 h, proliferation of LY294002-treated cells was significantly reduced, indicating sensitivity to the inhibition of PI3K activity. However, there was no difference among various concentrations of LY294002-treated cells at 12 h (Fig. 4a). After 24 h of treatment, proliferation of folate-deficient cells noticeably decreased with increased LY294002 concentrations (Fig. 4b, *p* < 0.001). Data at 36 and 48 h of LY294002 treatment were similar to those at 24 h (Fig. 4c). Conversely, no effect was observed in the growth patterns of PD98059-treated mESCs at different concentrations (Fig. 4d, *p* > 0.05). Additionally, cells treated with DMSO or LY294002 (25uM) for 48 h were harvested for western blot assays to detect AKT, FOXO1, and Bim protein levels to confirm the inactivation of AKT. Compared to the DMSO group, AKT protein levels were clearly reduced after LY294002 treatment (0.09 ± 0.01 vs 0.18 ± 0.02, *p* < 0.01), while FOXO1(0.74 ± 0.05 vs 0.35 ± 0.02, *p* < 0.001) and Bim protein levels(1.07 ± 0.20 vs 0.70 ± 0.05, *p* < 0.01) were increased significantly (Fig. 4e). Therefore, LY294002 obviously reduced cell proliferation in dose-dependent and time-dependent manners by inhibiting the PI3K/Akt/FOXO1/Bim signaling pathway.

### Bim is a putative direct target of miR-302a

**Fig. 5:**
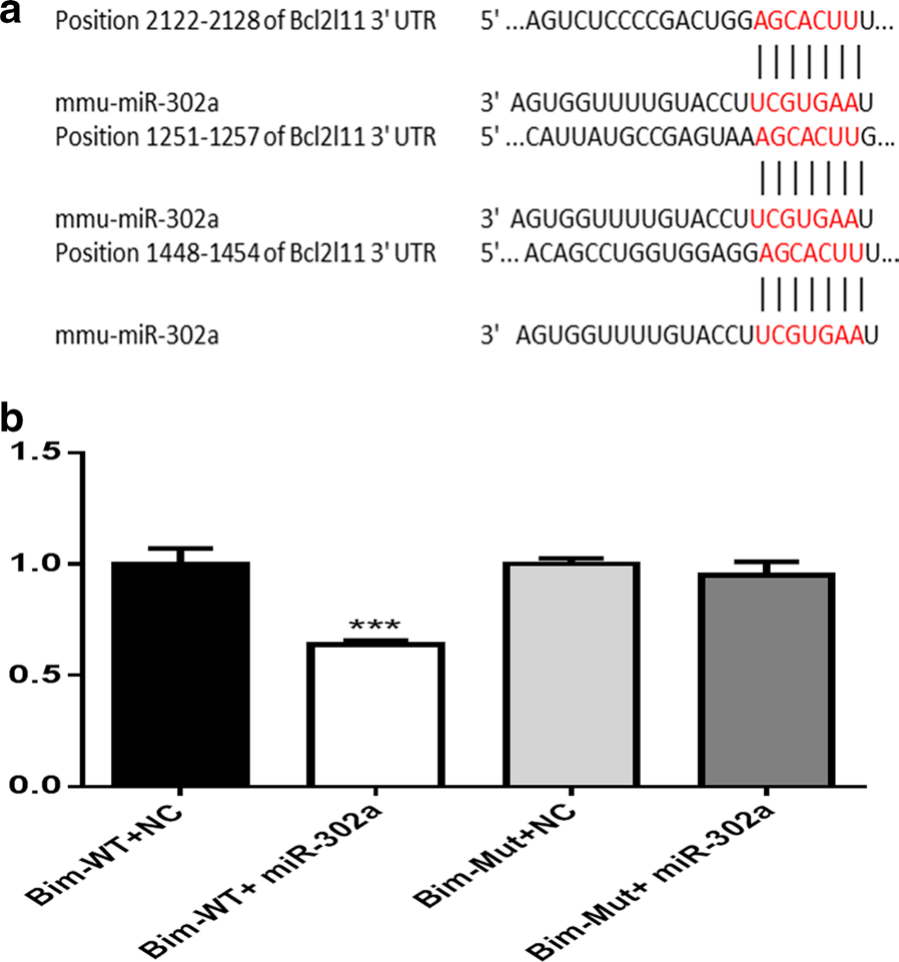
Luciferase activity assay and proposed signal pathways. **a** The three binding sites for miR-302a on Bim mRNA 3′-UTR. **b** Relative luciferase activity of reporter plasmids carrying wild-type or mutant Bim 3′UTR in cells cotransfected with negative control (NC) or miR-302a mimic, ***P < 0.001 versus other groups

Bim expression was inversely related to miR-302a expression; therefore, we tested whether miR-302a binds Bim or not by performing a bioinformatics search for candidate targets of miR-302a. The analysis indicated that there are three binding sites for miR-302a in the Bim 3′UTR: one well conserved site at 2122–2128 and two poorly conserved sites at 1251–1257 and 1448–1454 (Fig. 5a). The data showed that miR-302a suppressed luciferase activity by approximately 40% with the wild-type Bim 3′UTR (Fig. 5b, *p* < 0.01), but no significant suppression was observed with a mutant Bim 3′UTR. These results suggested that miR-302a binds directly to the predicted binding site(s) in the Bim 3′-UTR and negatively regulates Bim expression.

### Proposed FOXO1-miR-302a-Bim loop pathway initiates cell apoptosis in folate-deficient mESCs

**Fig. 6:**
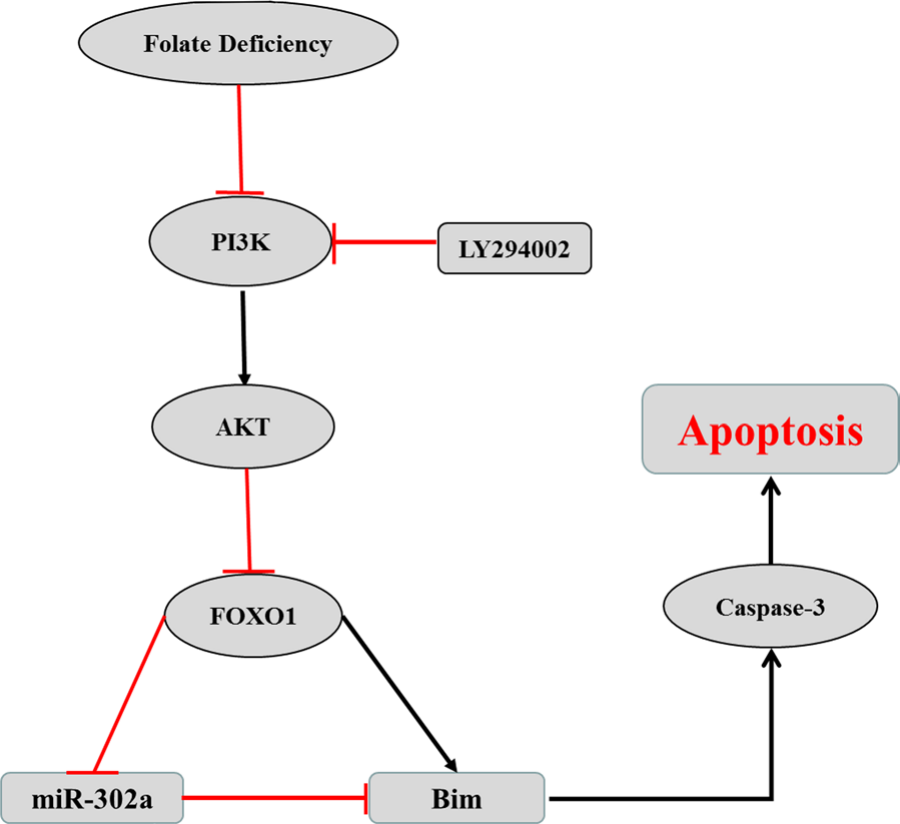
Simplified schematic presentation of the proposed signal pathways of folate deficiency on cell apoptosis of mESCs, from PI3K to apoptosis. Red T-shape arrow indicates inhibited; black common arrow indicates actived

These data led us to hypothesize there was a TF-miRNA-gene regulatory network, explaining how folic acid regulates cell apoptosis. When mESCs were cultured in folate-deficient conditions, the PI3K and AKT levels were repressed, which can negatively regulate the FOXO1 transcription factor. FOXO1 will translocate to the nucleus where it binds the Bim promoter, activating the pro-apoptosis gene Bim. FOXO1 was also confirmed to directly bind the miR-302a promoter region, inhibiting miR-302a activity. As the miR-302a level decreased, Bim expression was activated, in turn initiating apoptosis (Fig. 6). Nonetheless, knockdown or overexpression of FOXO1 may be indispensable to verify this hypothesis in the future.

## Discussion

An increasing amount of evidence suggests a relationship between folate status and miRNAs, as observed in many types of cell culture, such as mouse embryonic stem cells, mouse neural crest cells, human embryonic kidney cells, and cancer cell lines, as well as some human cohorts [6, 7, 15]. Folate and miRNA interact in a bi-directional nature: folate status modulates miRNA profiles, while miRNAs are involved in the regulation of the one-carbon cycle [6]. The biogenesis of miRNA is regulated at multiple levels, including during transcription and processing by Drosha and Dicer in the nucleus and cytoplasm, respectively [15]. Most miRNAs and TFs that participate in feedback loops are highly connected: miRNAs regulate and are regulated by many TFs, and vice versa [16]. This is the first study to address the involvement of TFs in the relationship between folate deficiency and miRNAs. We ranked miRNAs and TFs according to their degree of correlation and annotated whether they participate in a loop or not. The resulting TF-miRNA-gene network, together with previous miRNA-GO networks, suggested that FOXO1 and miR-302a contributed the most to the regulation network. In order to avoid both biological and technical noise and provide good accuracy and coverage, TF-miRNA interactions were verified by in vivo or in vitro chromatin immunoprecipitation (ChIP) using methods based on binding of large pools of DNA fragments. To answer this question, we performed a ChIP assay to determine whether FOXO1 binds to the miR-302a gene locus. The data confirmed that the transcription factor FOXO1 is capable of binding to the mouse miR-302a promoter region in a time dependent manner. However, ChIP enrichment of miR-302a was inhibited at 24 h, which was inconsistent with the qRT-PCR assays. Several reasons may explain these results. First, it was reasonable that altered miRNAs may be early sensors and in response to nutrient stress, especially miR-302a. The intracellular folate concentration was measured at 24 h intervals after folate-starvation began. However, it did not decrease significantly within the first 24 h infolate-deficient medium [7]. ChIP enrichment of miR-302a may be related to the intracellular folate concentration, as the folic acid may alter genomic methylations and affected the binding between FOXO1 and miR-302a. Methylated DNA immunoprecipitation combined with qPCR (MeDIP-qPCR) will be performed in our future research. Second, there were many steps during ChIP process. In particular, there were 28 binding regions, and we only selected the 904–916 bp promoter region with the highest score. Due to the discrepancy between the software prediction and the actual PCR results, it may be better to design multiple pairs of primers and reduce the false positive and false negative rates. Third, ChIP-qPCR is a perfect combination of ChIP technology and real-time quantitative PCR, with minimal cost. However, ChIP is a low-efficiency assay, and many TFs are not sufficiently broadly or highly expressed. Therefore, the high-throughput technique, such as ChIP-sequence and ChIP-chip may be required for studying the interaction between DNA and protein in vivo.

Members of the FoxO family were first identified in *C. elegans* as the ortholog DAF-16 [17], which consisted of 19 sub-families that share a highly conserved DNA-binding domain of approximately 110 amino acids [18]. FOXOs may act as transcriptional factors by inducing the expression of target genes with FOXO response elements. The core DNA recognition motif TTGTTTAC is recognized by all FOXO family members [19]. Lung cancer stem cells may arise from a microenvironment with low folate exposure through the activation of an Akt-mTOR-HIF1-FOXO3a signaling network [20]. However, little is known as to whether FOXO1 participates in folate deficiency-induced cell apoptosis. We predicted that FOXO1 might bind to miRNAs, such as let-7a, miR-124A, miR-15a, miR-15b, miR-16, miR-29b, miR-302a, and miR-34a. In particular, we determined that FOXO1 can bind directly to the miR-302a promoter region. Folate-free mESCs promotes the binding of FOXO1 to the miR-302a promoter, resulting in decreased miR-302a expression, indicating that miR-302a is a novel FOXO1 target. One gene can be targeted by multiple miRNAs, and in turn one miRNA can also regulate multiple target genes. There are much more complicated interaction networks among FOXO1 and ESC-specific miRNAs under folate deficient conditions. Acetylation, phosphorylation, and ubiquitination regulate FOXO1 activity by altering its subcellular localization [21]. In the present study, folate deficiency altered FOXO1 phosphorylation at the site of serine 256, which was preferentially phosphorylated through the Akt pathway.

AKT, a key factor and important antiapoptotic regulator, it was one of the most important proteins in the downstream signaling pathways of PI3K [22]. FOXO1 was also regarded as a key direct effector of PI3K/AKT signaling pathway [23]. To determine whether folate deficiency altered cell proliferation and apoptosis was mediated via the PI3K/Akt/FOXO1 pathway or others, and thus, the PI3K/AKT pathway inhibitor LY294002 and MAPK-specific inhibitor PD98059 were selected. A CCK-8 assay confirmed that cell proliferation in samples treated with LY294002 was obviously inhibited in dose-dependent and time-dependent manners. These findings indicated the PI3K/Akt signaling pathway was the main regulator of FoxO1, consistent with other studies on oocyte maturation and preimplantation embryo development in mice [24]. It seems that folate deficiency promoted transcriptional activation of FOXO by decreasing the level of phosphorylated AKT in a time-dependent manner, contributing to the induction of apoptosis. These results are also consistent with findings from other studies showing that inhibition of AKT led to activation of FOXO1, in turn initiating apoptosis [25, 26]. FOXO proteins function predominantly as TFs in the nucleus and bind as monomers to their cognate DNA-targeting sequences [27]. FOXO1 downstream targets, such as Puma, Bim, Fas ligand (FasL) p21, p27, PTEN and cyclins, significantly inhibited cell proliferation and promoted apoptosis and cell cycle arrest [28, 29]. Bim is an important pro-apoptotic molecule that induces cell apoptosis by interacting with anti-apoptotic proteins. Two conserved FOXO binding sites have been located in the Bim promoter, which are required for activation of the Bim promoter [30, 31]. FOXO transcription factors directly activate bim gene expression and promote apoptosis in sympathetic neurons, hematopoietic cells and breast cancer [30, 32, 33]. Under folate-free conditions, FOXO1 may induce expression of Bim triggered programmed cell death in mESCs. Thus, we are the first to report that Bim is a novel direct target of miR-302a. The results of this study support our conclusion that miR-302a regulates Bim. First, TargetScan predicted functional binding sites for miR-302a in the Bim 3′UTR. Second, miR-302a suppressed luciferase activity when the luciferase reporter plasmid carried the wild-type Bim 3′UTR (*p* < 0.001), but not in other groups. Functional assays will need to be performed to study Bim mRNA and protein expression after transfection with miR-302a mimics or treatment with inhibitors.

Placing miR302a between FOXO1 and Bim was the most novel contribution of this research. It was a classical mechanism that FOXO1 directly activated Bim expression. The present data provided some evidence that FOXO1 may regulate Bim expression by controlling the expression of miR-302a that regulated Bim expression levels. MiR-302a may participate as a regulatory layer in an alternative pathway. In the future, additional experiments will be needed to confirm whether down regulation of miR302a by FOXO1 is required for the effect of FOXO1 on Bim.

## Conclusions

This study provided evidence that the effects of folate deficiency on mammalian development may be mediated by the FOXO1-miR-302a-Bim loop that regulated cell apoptosis in mESCs. The involvement of miR-302a in folate deficiency-induced apoptosis through the AKT-FOXO1-BIM pathway in mESCs is a unique demonstration of the regulation mechanism of nutrient expression in embryonic development. Therefore, this study provides an epigenetic basis for folic acid supplementation during pregnancy.

## Data Availability

All data generated and analysed during the current study are available from the corresponding author on reasonable request.

## Abbreviations

miR: MicroRNA
mESCs: Mouse embryonic stem cells
hESCs: Human embryonic stem cells
TF: Transcription factor
FOXO: Forkhead box-containing protein, O subfamily
GO: Gene ontology
ChIP-qPCR: Chromatin immunoprecipitation-quantitative polymerase chain reaction
